# Exposure to low-dose perfluorooctanoic acid promotes hepatic steatosis and disrupts the hepatic transcriptome in mice

**DOI:** 10.1016/j.molmet.2022.101602

**Published:** 2022-09-14

**Authors:** Brecht Attema, Aafke W.F. Janssen, Deborah Rijkers, Evert M. van Schothorst, Guido J.E.J. Hooiveld, Sander Kersten

**Affiliations:** 1 Nutrition, Metabolism and Genomics Group, Division of Human Nutrition and Health, Wageningen University, the Netherlands; 2 Wageningen Food Safety Research (WFSR), Wageningen, the Netherlands; 3 Human and Animal Physiology, Wageningen University, the Netherlands

**Keywords:** PFOA, GenX, PPARα, Lipid metabolism, Dyslipidemia, NAFLD, CAR, constitutive androstane receptor, EDCs, endocrine-disrupting chemicals, NAFLD, non-alcoholic fatty liver disease, NEFA, non-esterified fatty acids, PCN, pregnenolone 16α-carbonitrile, PFAS, perfluoroalkyl substances, PFOA, perfluorooctanoic acid, PFOS, perfluorooctanesulfonic acid, PPARα, peroxisome proliferator-activated receptor alpha, PXR, pregnane X receptor, SREBP, sterol regulatory element-binding protein

## Abstract

**Objective:**

Perfluoroalkyl substances (PFAS) are man-made chemicals with demonstrated endocrine-disrupting properties. Exposure to perfluorooctanoic acid (PFOA) has been linked to disturbed metabolism via the liver, although the exact mechanism is not clear. Moreover, information on the metabolic effects of the new PFAS alternative GenX is limited. We examined whether exposure to low-dose PFOA and GenX induces metabolic disturbances in mice, including NAFLD, dyslipidemia, and glucose tolerance, and studied the involvement of PPARα.

**Methods:**

Male C57BL/6J wildtype and PPARα^−/−^ mice were given 0.05 or 0.3 mg/kg body weight/day PFOA, or 0.3 mg/kg body weight/day GenX while being fed a high-fat diet for 20 weeks. Glucose and insulin tolerance tests were performed after 18 and 19 weeks. Plasma metabolite levels were measured next to a detailed assessment of the liver phenotype, including lipid content and RNA sequencing.

**Results:**

Exposure to high-dose PFOA decreased body weight and increased liver weight in wildtype and PPARα^−/−^ mice. High-dose but not low-dose PFOA reduced plasma triglycerides and cholesterol, which for triglycerides was dependent on PPARα. PFOA and GenX increased hepatic triglycerides in a PPARα-dependent manner. RNA sequencing showed that the effects of GenX on hepatic gene expression were entirely dependent on PPARα, while the effects of PFOA were mostly dependent on PPARα. In the absence of PPARα, the involvement of PXR and CAR became more prominent.

**Conclusion:**

Overall, we show that long-term and low-dose exposure to PFOA and GenX disrupts hepatic lipid metabolism in mice. Whereas the effects of PFOA are mediated by multiple nuclear receptors, the effects of GenX are entirely mediated by PPARα. Our data underscore the potential of PFAS to disrupt metabolism by altering signaling pathways in the liver.

## Highlights

• Exposure to PFOA in WT mice results in a reduction in plasma triglycerides and cholesterol.
• PFOA and GenX induce hepatic lipid accumulation in WT but not in PPARα^−/−^ mice.
• PFOA impacts liver gene expression predominantly via PPARα and to a minor extent via PXR and CAR.
• GenX is a specific activator of PPARα.

## 1 Introduction

Obesity and related metabolic disorders such as non-alcoholic fatty liver disease (NAFLD), type 2 diabetes, and dyslipidemia cause an ever-growing burden on our society [1]. With estimations of more than 39% of the human population being overweight worldwide [2], delineating the causes of these disorders is becoming of utmost importance. While diet, lifestyle, and genetics are well known to contribute to the development of obesity and related metabolic disorders [3], there are growing indications that exposure to certain chemicals in the environment may also play a role. These environmental chemicals originally gained attention due to their capability to interfere with normal endocrine function, hence labeling them as endocrine-disrupting chemicals (EDCs) [4]. Later on, a specific group of EDCs has been defined based on their impact on metabolism [5]. These substances, now commonly defined as obesogens or metabolism-disrupting chemicals, are known to affect metabolic processes within the body and may thereby contribute to obesity, NALFD, and type 2 diabetes [6].

A group of chemicals that has been associated with metabolic disturbances is perfluoroalkyl substances (PFAS). PFAS are man-made chemicals that are extensively used in industrial products due to their high-temperature resistance and water- and dirt-repellent properties. Accordingly, PFAS are present in a wide array of consumer products, including non-stick coatings, food packaging, and firefighting foams. The general structure of PFAS consists of a hydrophobic fluorinated alkyl chain of variable length joined to a hydrophilic end group [7]. PFAS generally exhibit long half-lives, causing them to accumulate both in the environment and the human body [7]. Perfluorooctanoic acid (PFOA), a well-known PFAS, has an estimated serum half-live of 2–4 years in humans [8,9].

Studies have shown that an important molecular target of PFAS, including PFOA, is the nuclear receptor Peroxisome Proliferator-Activated Receptor α (PPARα) [[10], [11], [12]]. PPARs form a group of nuclear receptors that play essential roles in the transcriptional regulation of lipid homeostasis. Three subtypes can be distinguished, consisting of PPARα, PPARβ/δ, and PPARγ, each of which is characterized by a different expression pattern and function. PPARα is particularly important in the liver [13], where it transcriptionally regulates numerous enzymes and factors involved in nearly every branch of lipid metabolism, including fatty acid oxidation, fatty acid uptake, and triglyceride turnover [14,15]. PPARα can be activated by endogenous ligands such as fatty acids and their eicosanoid derivatives, as well as by synthetic agonists such as fibrates [[16], [17], [18]]. PPARα agonists are used in the treatment of dyslipidemia and are being examined for their ability to ameliorate NAFLD, illustrating the importance of PPARα in lipid metabolism [19]. Besides fibrates, PFOA, which structurally resembles fatty acids, also potently activates mouse and human PPARα [10,20,21]. In mouse liver and human HepaRG cells, PFOA induces the expression of numerous PPARα target genes [[22], [23], [24]]. Although there is no doubt that PPARα is a key molecular target of PFOA, there is evidence that PFOA impacts hepatic lipid accumulation in the absence of PPARα, suggesting that additional molecular mechanisms likely play a role in the lipid disturbances triggered by PFOA [22,25].

In line with its ability to activate PPARα and its target genes, rodent data indicate that PFOA influences lipid homeostasis in the liver, which is the major target organ of PFOA [23,[26], [27], [28], [29], [30]]. Specifically, exposure to PFOA has been repeatedly shown to promote hepatic lipid accumulation in vivo [24,28,31,32]. In human HepaRG liver cells, exposure to PFOA also resulted in triglyceride accumulation and caused the downregulation of genes related to cholesterol biosynthesis [33,34]. Despite extensive research, the effects of PFOA on the development of obesity, glucose intolerance, and NAFLD have not yet been investigated.

Because of its suspected effects on human health, major efforts have been made to phase out the use of many PFAS. As a result, in 2019, more than 180 countries agreed to ban the production and use of PFOA. However, in response to the ban on PFOA, new replacement chemicals have been produced. 2,3,3,3-Tetrafluoro-2-(heptafluoropropoxy)propanoic acid (HFPO-DA or GenX, referring to the namesake technology) is an example of such a replacement [35]. Currently, there is very limited information on the metabolic effects of GenX in vivo. Recently, it was shown that the treatment of mice with GenX leads to the upregulation of many PPARα targets in the liver [36,37]. However, the overall impact of GenX on the development of obesity, glucose intolerance, and NAFLD has not been investigated. Also, the importance of PPARα in mediating the in vivo effects of GenX remains unclear. Accordingly, the present study aimed to examine the effect of PFOA and GenX on the development of obesity, glucose intolerance, and NAFLD. To that end, we used a model of diet-induced obesity in which mice were given a high-fat diet for 20 weeks concurrent with the provision of PFOA or GenX via drinking water. To investigate the role of PPARα, the experiments were run in parallel in wildtype C57BL/6J mice and their PPARα^−/−^ counterparts.

## 2 Material and methods

### 2.1 Animals

Male and female wildtype and PPARα^−/−^ mice that had been backcrossed on a pure C57BL/6J background for more than 10 generations were acquired from Jackson Laboratories (no. 000664 and 008154, respectively). The mice were further bred at the animal facility of Wageningen University under specific pathogen-free conditions to generate the number of mice necessary for the experiments. Animals were housed on a 12 h light–dark cycle with normal bedding and cage enrichment and held at the animal facility of Wageningen University.

At 9–11 weeks of age, male wildtype and PPARα^−/−^ mice received either PFOA or GenX via the drinking water while being fed a high-fat diet for 20 weeks (45% kcal fat; D12451, Research Diets, New Brunswick). PFOA (Perfluorooctanoic acid, CAS no. 335-67-1; purity 95%) was purchased from Sigma–Aldrich and GenX (2,3,3,3-Tetrafluoro-2-(heptafluoropropoxy)propanoic acid, CAS no. 13252-13-6; purity 97%) was purchased from Synquest laboratories (Alachua FL, US). PFOA was added to the drinking water to a concentration that was calculated to lead to an exposure of 0.05 or 0.3 mg/kg body weight/day. For GenX, a single concentration in the drinking water was used that was calculated to lead to an exposure of 0.3 mg/kg body weight/day. Three different treatment groups were thus included per genotype next to the control group, each containing 12 mice per group, leading to a total of 48 mice per genotype. The mice had ad libitum access to food and drinking water. Body weights, food intake, and water intake were assessed weekly.

After 20 weeks of exposure, mice were euthanized in the fed state at Zeitgeber time (ZT)2.5 – ZT4.5. Mice were anesthetized with isoflurane followed by blood collection via orbital puncture. Lean and fat mass was subsequently measured using EchoMRI 100 V (EchoMedical Systems, Houston, TX, USA). Immediately thereafter, the mice were euthanized by cervical dislocation, and tissues were collected. Tissues were weighed, prepared for histological analyses, or snap frozen in liquid nitrogen and subsequently stored at −80 °C. The animal study was approved by the central committee on animal experimentation and the local animal welfare committee of Wageningen University (AVD104002015236, 2016.W-0093.020).

#### 2.1.1 Intraperitoneal glucose and insulin tolerance test

Intraperitoneal glucose and insulin tolerance tests were performed after 18 and 19 weeks of treatment, respectively. For the glucose tolerance test, the mice fasted for 5 h, after which blood was collected via tail bleeding for baseline blood glucose measurement (t = 0). Next, the mice received an intraperitoneal injection of 0.8 mg/kg body weight glucose (ThermoFisher Scientific, MA, USA), followed by blood collection via tail bleeding at 15, 30, 45, 60, 90, 120, and 150 min. For the insulin tolerance test, the mice fasted for 5 h, after which blood was collected via tail bleeding for baseline blood glucose measurement (t = 0). Next, the mice received an intraperitoneal injection of 0.75 U/kg body weight insulin (Actrapid; Novo Nordisk A/S, Denmark), followed by blood collection at 15, 30, 45, 60, and 90 min. Glucose levels in blood were measured with GLUCOFIX Tech glucometer and glucose sensor test strips (Menarini Diagnostics, Valkenswaard, The Netherlands).

### 2.2 Plasma measurements

Blood was collected in EDTA tubes (Sarstedt, Nümbrecht, Germany) and spun for 15 min at 5.000 RPM at 4 °C. Plasma aliquots were made and stored at −80 °C before subsequent analyses. Plasma triglycerides (Liquicolor Mono, Human GmbH, Wiesbaden, Germany), cholesterol (Cholesterol FS assay, DiaSys Diagnostic Systems GmbH, Holzheim, Germany), non-esterified fatty acids (NEFA) (Instruchemie, Delfzijl, the Netherlands), glycerol (Instruchemie), glucose (Glucose GOD FS 10′, DiaSys), and β-hydroxybutyrate (Sigma–Aldrich) were measured according to the instruction of the manufacturers.

### 2.3 Liver triglycerides and glycogen

For measurement of liver triglycerides, 5% liver homogenates were made in a buffer containing sucrose (250 mM), EDTA (2 mM), Tris–base (10 mM) at pH 7.5. Triglycerides were subsequently measured using a commercially available kit (Liquicolor mono) according to the instruction of the manufacturers.

To measure glycogen, liver pieces (approximately 50 mg) were dissolved in 10 volumes of 1 M NaOH and incubated at 55 °C for 1–2 h. Afterward, an equal volume of 1 M HCL was added, followed by a 5 min centrifugation step at 3,000 RPM. Next, amyloglucosidase (1000 U/ml in 0.2 M sodium acetate 4.8 pH) was added to the sample (1:10 ratio) in order to break down glycogen into glucose. The mixture was incubated for 2 h at 42 °C while shaking at 700 RPM, after which the samples were centrifuged shortly. Glucose levels were subsequently measured by the use of a commercially available kit (Glucose GOD FS 10′, DiaSys).

### 2.4 Liver histology

Fresh liver tissue was fixed in 4% paraformaldehyde, dehydrated, and embedded in paraffin. Thin sections of the samples were prepared at 5 μm using a microtome and placed onto glass slides followed by overnight incubation at 37 °C. Liver sections were stained with hematoxylin & eosin (H&E). To this end, liver tissues were stained in Mayer hematoxylin solution for 10 min and eosin for 10 s at room temperature with intermediate washing in ethanol. The tissues were allowed to dry at room temperature and subsequently imaged using light microscope.

### 2.5 HepaRG experiments

The human hepatic cell line HepaRG was obtained from Biopredic International (Rennes, France) and cultured in growth medium consisting of William's E Medium + GlutaMAX™ (Thermofisher Scientific, Landsmeer, The Netherlands) supplemented with 10% Good Forte filtrated bovine serum (FBS; PAN™ Biotech, Aidenbach, Germany), 1% PS (100 U/ml penicillin, 100 μg/ml streptomycin; Capricorn Scientific, Ebsdorfergrund, Germany), 50 μM hydrocortisone hemisuccinate (sodium salt) (Sigma–Aldrich), and 5 μg/ml human insulin (PAN™ Biotech).

HepaRG cells were seeded in 24-well plates (Corning, Corning, NY; 55,000 cells per well in 500 μl) according to the HepaRG instruction manual from Biopredic International. After 2 weeks on growth medium, cells were cultured for two days in growth medium supplemented with 0.85% DMSO to induce differentiation. Subsequently, cells were cultured for 12 days in growth medium supplemented with 1.7% DMSO (differentiation medium) for final differentiation. At this stage, cells were ready to be used for toxicity studies. Cell cultures were maintained in an incubator (humidified atmosphere with 5% CO2 at 37 °C) and the medium was refreshed every 2–3 days during culturing. Prior to the toxicity studies, differentiated HepaRG cells were incubated for 24 h in assay medium (growth medium-containing 2% FBS) supplemented with 0.5% DMSO. Differentiated HepaRG cells were subsequently exposed for 24 h to PFOA and GenX in different concentrations up to 400 μM.

### 2.6 RNA isolation and quantitative PCR

To isolate RNA from the liver, tissues were homogenized using TRIzol reagent (Life Technologies, Bleiswijk, The Netherlands). To isolate RNA from human HepaRG cells, RLT buffer was used. RNA was subsequently isolated and purified by using the RNeasy mini kit from Qiagen (Venlo, The Netherlands). RNA concentration was measured with Nanodrop 1000 spectrophotometer and for subsequent quantitative PCRs, 500 ng RNA was used as input to synthesize cDNA by using iScript cDNA synthesis kit (Bio-rad Laboratories, Veenendaal, The Netherlands). Gene expression was measured by using Sensimix (Bioline, GC Biotech, Alphen aan den Rijn, The Netherlands) on a CFX384 real-time PCR detection system (Bio-Rad Laboratories, Veenendaal, the Netherlands). Gene expression data were normalized to Cyclophilin for mouse tissues and to RPL27 for the HepaRG cells. A list of primer sequences is presented in Table 1.

**Table 1:** List with primers for qPCR.

Name	Forward	Reverse
*mCyclophilin*	CAGACGCCACTGTCGCTTT	TGTCTTTGGAACTTTGTCTGCAA
*mCd36*	AGATGACGTGGCAAAGAACAG	CCTTGGCTAGATAACGAACTCTG
*mCyp4a14*	AGGCAGTCCAATTCTACTTACG	GCTCCTTGTCCTTCAGATGG
*mEhhadh*	AAAGCTAGTTTGGACCATACGG	ATGTAAGGCCAGTGGGAGATT
*mLpl*	CAGCTGGGCCTAACTTTGAG	GACCCCCTGGTAAATGTGTG
*mFgf21*	CTGCTGGGGGTCTACCAAG	CTGCGCCTACCACTGTTCC
*hRPL27*	ATCGCCAAGAGATCAAAGATAA	TCTGAAGACATCCTTATTGACG
*hFABP4*	ACTGGGCCAGGAATTTGAC	GCATTCCACCACCAGTTTATC
*hPLIN2*	ATGGCATCCGTTGCAGTTGAT	GATGGTCTTCACACCGTTCTC

### 2.7 RNA sequencing

For RNA sequencing on mouse liver, 4 mice per group were used. Total RNA from liver was isolated as stated above. RNA integrity was determined using an Agilent 2100 Bioanalyzer with RNA 6000 microchips (Agilent Technologies, Santa Clara, CA). Library construction and RNA sequencing runs on the BGISEQ-500 platform [38] were conducted at Beijing Genomics Institute (BGI, Hong Kong). At BGI, Genomic DNA was removed with two digestions using Amplification grade DNAse I (Invitrogen, USA). The RNA was sheared and reverse transcribed using random primers to obtain cDNA, which was used for library construction. The library quality was determined using a Bioanalyzer 2100. Thereafter, the library was used for 100bp paired-end sequencing on the sequencing platform BGISEQ-500 (BGI). All the generated raw sequencing reads were filtered by removing reads with adaptors, reads with more than 10% of unknown bases, and low-quality reads. Clean reads were then obtained and stored in FASTQ format.

#### 2.7.1 Processing of RNA sequencing reads

The RNA-seq reads were used to quantify transcript abundances. The tool *Salmon* [39] (version 1.5.1) was used to map the reads to the GRCm39 mouse genome assembly-based transcriptome sequences as annotated by the GENCODE consortium [40] (release M27). The obtained transcript abundance estimates and lengths were imported in R using the package *tximport* [41] (version 1.22.0), scaled by average transcript length and library size, and summarized at the gene-level. Differential gene expression was determined using the package *limma* [42] (version 3.50.0) utilizing the obtained scaled gene-level counts. Briefly, before statistical analyses, nonspecific filtering of the count table was performed to increase detection power [43], based on the requirement that a gene should have an expression level greater than 10 counts, i.e. ∼0.50 count per million reads (cpm) mapped, for at least 4 libraries across all 32 samples. Differences in library size were adjusted by the trimmed mean of M-values normalization method [44], implemented in the package *edgeR* [45] (version3.36.0). Counts were transformed to log2 (cpm) values and associated precision weights and entered into the *limma* analysis pipeline [46]. Differentially expressed genes were identified by using generalized linear models that incorporate empirical Bayesian methods [42,47]. Genes were defined as significantly changed when P ≤ 0.001 and fold change >1.5. RNA-seq data have been deposited to Gene Expression Omnibus (GEO) under accession number GSE212294.

#### 2.7.2 Biological interpretation of transcriptome data

Changes in gene expression were related to biologically meaningful changes using gene set enrichment analysis (GSEA) [48]. GSEA evaluates gene expression at the level of gene sets that are based on prior biological knowledge, e.g. published information about biochemical pathways or signal transduction routes, allowing more reproducible and interpretable analysis of gene expression data. As no gene selection step (fold change and/or p-value cut-off) is used, GSEA is an unbiased approach. Gene sets were retrieved from the expert-curated KEGG pathway database [49]. Only gene sets comprising more than 15 and fewer than 500 genes were taken into account. The statistical significance of GSEA was determined using 10,000 permutations.

### 2.8 Statistics

Data are presented as mean ± SEM. Statistical significance of treatment versus control was determined by two-way ANOVA with Dunnett multiple comparisons test. For statistical testing of wildtype versus PPARα^−/−^ mice, two-way ANOVA with Šídák’s multiple comparisons test was used. A value of p < 0.05 was considered as statistically significant. Data were visualized and analyzed using Prism version 9.0 (GraphPad Software, San Diego, CA, USA).

## 3 Results

**Figure 1:**
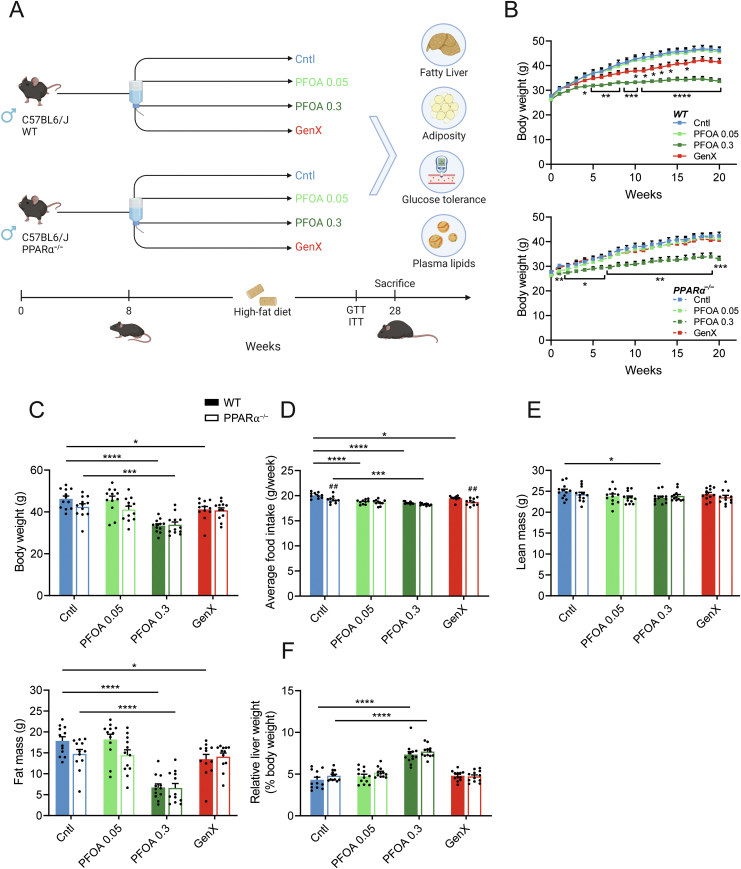
**High-dose PFOA reduces body weight and increases liver weight after 20 weeks of exposure independent of PPARα**. (A) Study design, created with Biorender. (B) Body weight trajectory during 20 weeks of exposure to 0.05 or 0.3 mg/kg bw/day PFOA, 0.3 mg/kg bw/day GenX, or control in wildtype or PPARα^−/−^ mice. (C) Body weights after 20 weeks. (D) Average food intake per week during week 5–15 of treatment. (E) Lean and fat mass as determined by EchoMRI. (F) Liver weight relative to body weight. Graphs are presented as mean ± SEM (n = 10–12 mice per group). Asterisks indicate significant differences between treatment vs control ∗p < 0.05, ∗∗p < 0.01, ∗∗∗p < 0.001, ∗∗∗∗p < 0.0001. Hashtags indicate significant differences between wildtype vs PPARα^−/−^ mice within one treatment group (^##^p < 0.01).

In the current study, we set out to better understand the potential metabolism-disrupting effects of PFOA and GenX in a mouse model of obesity, glucose intolerance, and NAFLD. To this end, C57BL/6J mice fed a high-fat diet were exposed to PFOA or GenX in the drinking water for 20 weeks (Figure 1A). Mice were exposed to lower doses of PFOA or GenX than in previous rodent studies in order to better relate the findings to human exposure levels (0.05 and 0.3 mg/kg body weight/day for PFOA, 0.3 mg/kg body weight/day for GenX [22,24,28,31,50]. In addition, to further explore the role of PPARα in mediating the potential metabolism-disrupting effects of PFOA and GenX, the study was conducted in wildtype and PPARα^−/−^ mice.

### 3.1 PFOA decreases body weight in wildtype and PPARα^−/−^ mice

During the 20 weeks of high-fat feeding, high-dose PFOA treatment significantly reduced weight gain in wildtype and PPARα^−/−^ mice. (Figure 1B, C). By contrast, treatment with GenX only reduced weight gain in the wildtype but not in the PPARα^−/−^ mice. Exposure to PFOA or GenX resulted in a significant reduction in food intake in the wildtype mice, consistent with the anorexic effects of known PPARα agonists [[51], [52], [53]] (Figure 1D). The changes in body weight in the mice exposed to GenX and high-dose PFOA were accompanied by a significant reduction in fat mass, but not lean mass (Figure 1E). Liver weights were significantly increased in the mice exposed to high-dose PFOA as compared to the control mice, irrespective of genotype (Fig. 1F).

**Figure 2:**
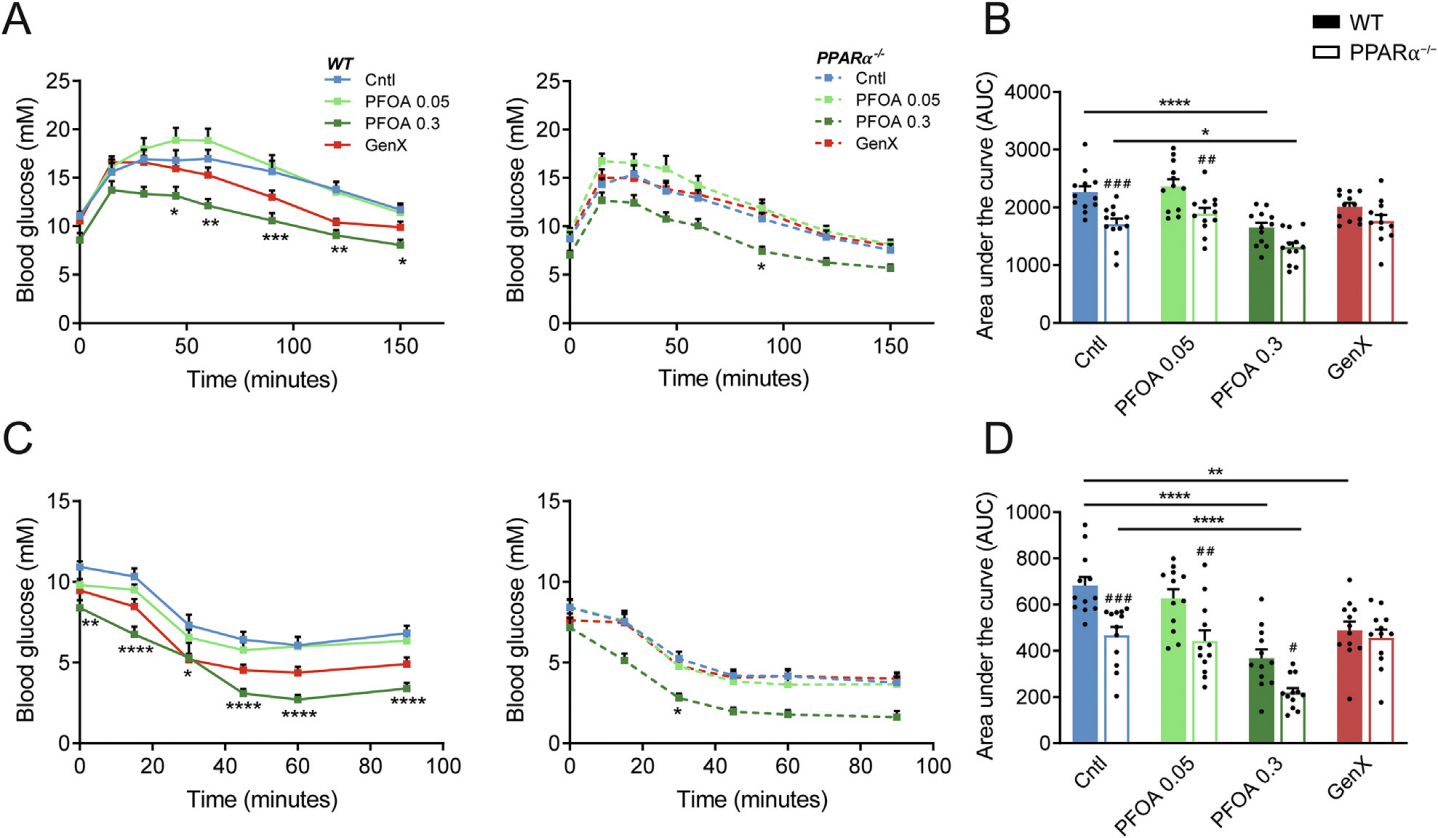
**Glucose and insulin tolerance are improved by high-dose PFOA in wildtype and PPARα**^**−/−**^**mice.** (A) Intraperitoneal glucose tolerance test (0.8 g glucose/kg body weight) after 18 weeks of treatment and (B) area under the curve. (C) Intraperitoneal insulin tolerance test (0.75 U insulin/kg body weight) after 19 weeks of treatment and (D) area under the curve. Graphs are presented as mean ± SEM (n = 6–12 mice per group). Asterisks indicate significant differences between treatment vs control (∗p < 0.05, ∗∗p < 0.01, ∗∗∗p < 0.001 ∗∗∗∗p < 0.0001). Hashtags indicate significant differences between wildtype vs PPARα^−/−^ mice within one treatment group (^#^p < 0.05, ^##^p < 0.01, ^###^p < 0.001, ^####^p < 0.0001).

To assess if the PFOA and GenX treatment might affect glucose tolerance and insulin sensitivity, glucose and insulin tolerance tests were performed. Overall, PPARα^−/−^ mice displayed increased glucose tolerance as compared to wildtype mice (Figure 2A, B), as well as increased insulin tolerance (Figure 2C, D). In the wildtype and PPARα^−/−^ mice, high-dose PFOA significantly improved glucose and insulin tolerance. By contrast, the low-dose PFOA and GenX treatments did not significantly impact glucose and insulin tolerance.

### 3.2 High-dose PFOA reduces plasma triglycerides and cholesterol

**Figure 3:**
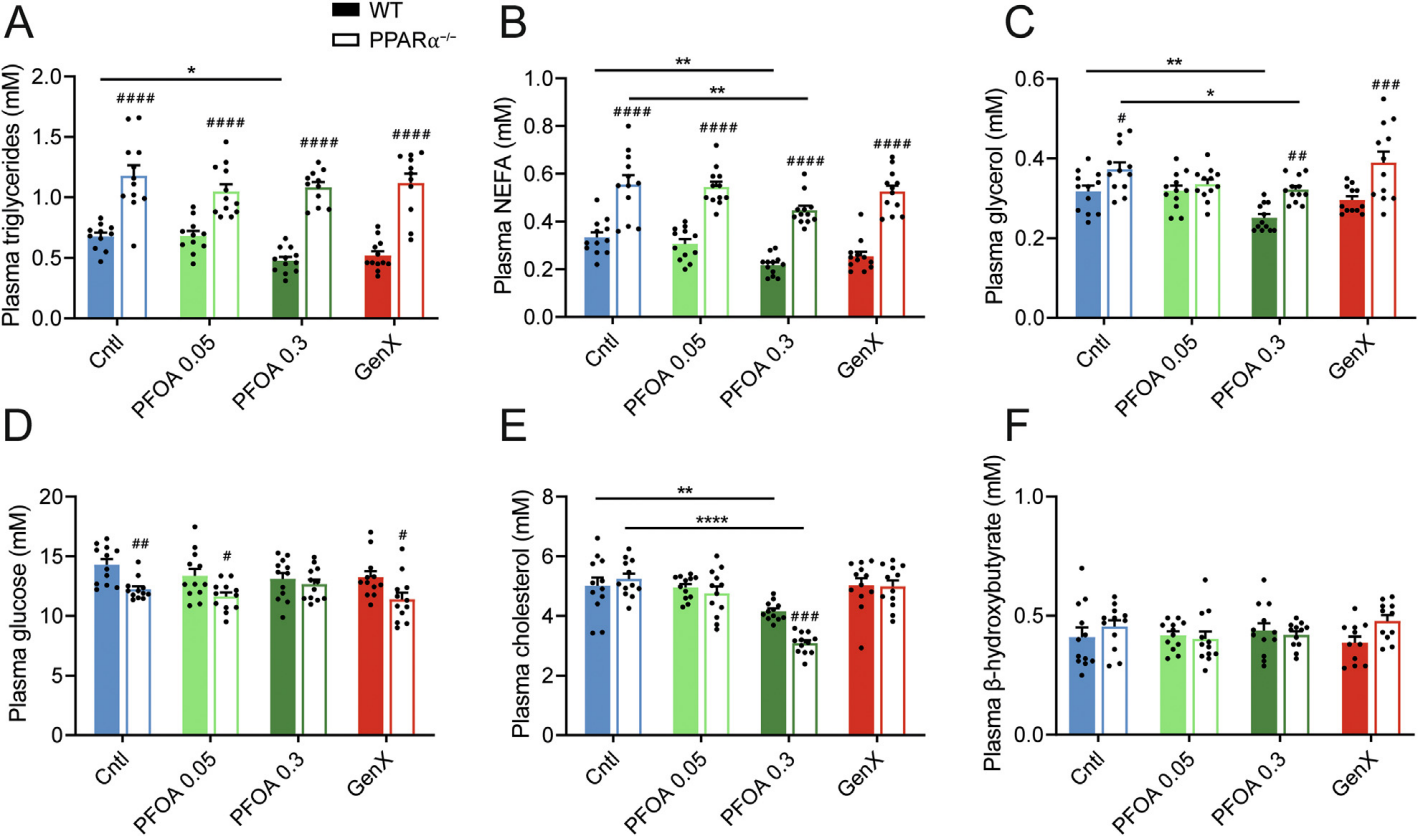
**Effect of PFOA and GenX on plasma metabolite levels in wildtype and PPARα**^**−/−**^**mice.** (A) Plasma triglycerides. (B) Plasma free fatty acids (FFA, NEFA). (C) Plasma glycerol. (D) Plasma glucose. (E) Plasma cholesterol. (F) Plasma β-hydroxybutyrate. Graphs are presented as mean ± SEM (n = 11–12 mice per group). Asterisks indicate significant differences between treatment vs control (∗p < 0.05, ∗∗p < 0.01, ∗∗∗p < 0.001 ∗∗∗∗p < 0.0001). Hashtags indicate significant differences between wildtype vs PPARα^−/−^ mice within one treatment group (^#^p < 0.05, ^##^p < 0.01, ^###^p < 0.001, ^####^p < 0.0001).

Because several studies have linked PFAS exposure to changes in levels of plasma triglycerides and cholesterol [9,23,27,50,54], we assessed plasma triglycerides, cholesterol, and other metabolites in the mice exposed to PFOA and GenX. As previously shown [55], ablation of PPARα was in general associated with significantly higher plasma triglycerides, NEFA, and glycerol levels, and significantly lower plasma glucose levels (Figure 3A–D). Treatment with high-dose PFOA significantly reduced plasma triglyceride levels in the wildtype mice but not the PPARα^−/−^ mice (Figure 3A). High-dose PFOA also significantly decreased plasma cholesterol levels, which was more pronounced in the PPARα^−/−^ mice than in the wildtype mice (Figure 3E). Furthermore, high-dose PFOA significantly decreased plasma NEFA and glycerol levels, which was observed in both wildtype and PPARα^−/−^ mice (Figure 3B, C). By contrast, treatment with low-dose PFOA or GenX did not significantly alter plasma triglycerides, cholesterol, NEFA, and glycerol levels in either wildtype or PPARα^−/−^ mice. None of the treatments significantly changed plasma glucose or β-hydroxybutyrate levels in either wildtype or PPARα^−/−^ mice (Figure 3D, F). Taken together, these data indicate that treatment with high-dose PFOA but not GenX or low-dose PFOA significantly reduced plasma triglycerides, cholesterol, NEFA, and glycerol levels, which was independent of PPARα.

### 3.3 Hepatic lipid metabolism is affected in PFOA and GenX treated mice

**Figure 4:**
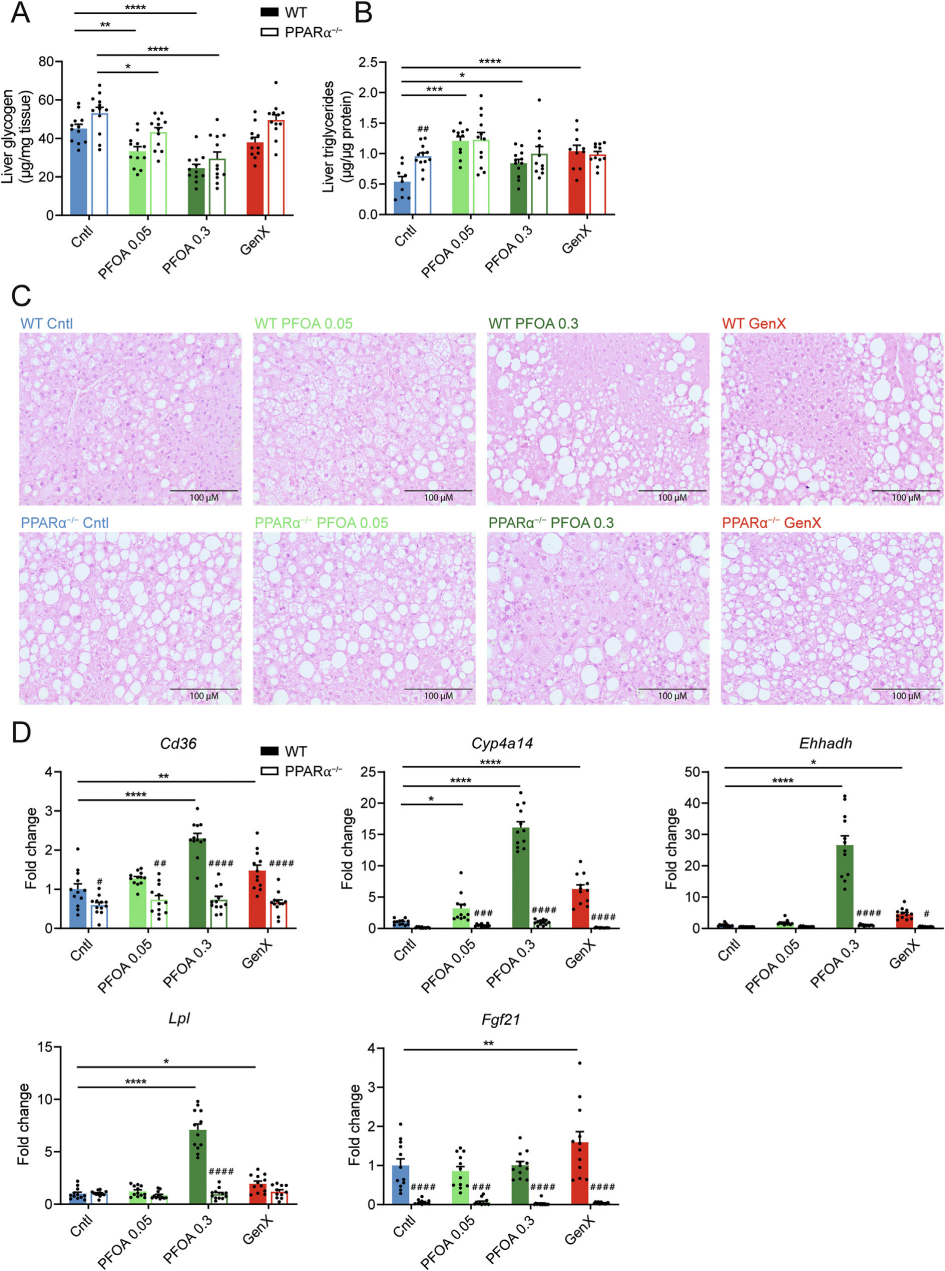
**Effect of PFOA and GenX on hepatic metabolism in wildtype and PPARα**^**−/−**^**mice.** (A) Glycogen concentrations in liver. (B) Triglyceride concentrations in liver. (C) H&E staining of representative liver sections (20× magnification). (D) Hepatic gene expression of *Cd326, Cyp4a14, Ehhadh, Lpl,* and *Fgf21.* Data are normalized to cyclophilin and expressed relative to wildtype control. Graphs are presented as mean ± SEM (n = 9–12 mice per group). Asterisks indicate significant differences between treatment vs control (∗p < 0.05, ∗∗p < 0.01, ∗∗∗p < 0.001 ∗∗∗∗p < 0.0001). Hashtags indicate significant differences between wildtype vs PPARα^−/−^ mice within one treatment group (^#^p < 0.05, ^##^p < 0.01, ^###^p < 0.001, ^####^p < 0.0001).

The liver is likely the main target organ of many types of PFAS [22,25,28,32]. As we found increased liver weight in the mice exposed to high-dose PFOA, we next set out to further assess the effects of PFOA and GenX on relevant metabolic parameters in the liver. Liver glycogen levels were elevated in PPARα^−/−^ mice compared to the wildtype mice (Figure 4A). Next to this, treatment with low- and high-dose PFOA significantly reduced liver glycogen levels, which was seen in both wildtype and PPARα^−/−^ mice. In agreement with previous studies [14,56], liver triglyceride content was significantly higher in untreated PPARα^−/−^ mice than in untreated wildtype mice (Figure 4B). Exposure to PFOA or GenX significantly increased liver triglyceride content in wildtype mice, which was abolished in PPARα^−/−^ mice (Figure 4B), suggesting that PPARα mediates the induction in liver triglycerides by PFOA and GenX. H&E staining was performed to examine the histology of the liver in the various treatment groups. In line with the quantitative analysis of triglyceride content, lipid droplet accumulation was higher in untreated PPARα^−/−^ mice than in untreated wildtype mice (Figure 4C). Treatment with PFOA or GenX noticeably increased lipid accumulation, which was abolished in the PPARα^−/−^ mice. Taken together, these data suggest that exposure to PFOA or GenX increased hepatic lipid accumulation in a PPARα-dependent manner.

PFOA is known to activate PPARα [18,20]. Consistent with this notion, high-dose PFOA significantly increased the hepatic expression of the classical PPARα target genes *Cd36, Cyp4a14, Ehhadh, and Lpl* (Figure 4D), while low-dose PFOA only increased the expression of *Cyp4a14*. Similarly, treatment with GenX significantly induced hepatic *Cd36*, *Cyp4a14*, *Ehhadh, and Lpl* mRNA. Remarkably, hepatic mRNA expression of endocrine factor *Fgf21* was increased only after treatment with GenX in the wildtype mice. The stimulation of PPARα target gene expression by PFOA and GenX was abolished in the PPARα^−/−^ mice, suggesting that the effects of PFOA and GenX on the above genes are mediated by PPARα.

### 3.4 PFOA and GenX induce distinct effects on the hepatic transcriptome in PPARα^−/−^ and wildtype mice

**Figure 5:**
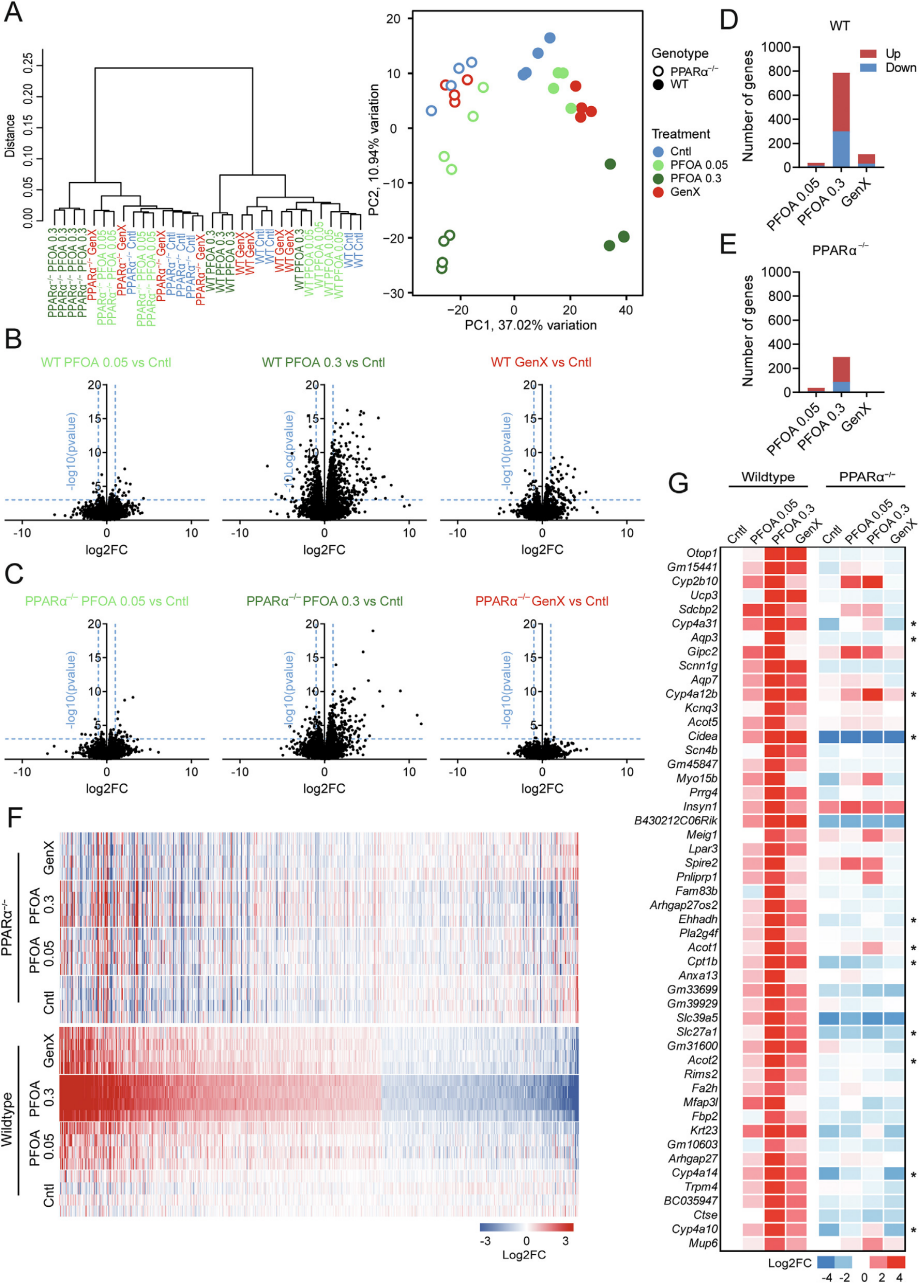
**Transcriptome effects of PFOA and GenX in livers of wildtype and PPARα^−/−^ mice.** (A) Hierarchical clustering and principle component analysis of transcriptome data from livers of wildtype or PPARα^−/−^ mice exposed to 0.05 or 0.3 mg/kg bw/day PFOA, GenX or control group. Volcano plot analysis of 0.05 or 0.3 mg/kg bw/day PFOA or GenX vs control in wildtype (B) or PPARα^−/−^ (C) mice. Total number of up- and downregulated genes (p ≤ 0.001 and FC >1.5) in wildtype (D) or PPARα^−/−^ (E) mice. (F) Heatmap based on significantly regulated genes (p < 0.001 and FC >1.5) of 0.3 mg/kg bw/day PFOA vs control in wildtype mice. (G) Heatmap of top 50 most highly upregulated genes by 0.3 mg/kg bw/day PFOA vs control in wildtype mice, (P ≤ 0.001). Asterisks indicate PPARα target genes.

To obtain a more global view of the effects of PFOA and GenX on hepatic gene expression and to further examine the role of PPARα, we performed RNA sequencing on all groups, using 4 biological replicates per group. First, hierarchical clustering and principal component analysis (PCA) were performed to assess the global transcriptomic changes in wildtype and PPARα^−/−^ mice after exposure to PFOA or GenX (Figure 5A). As expected, hierarchical clustering and PCA plots revealed clear clustering based on genotype. In addition, whereas the mice that received GenX or low-dose PFOA did not form distinct clusters in either wildtype or PPARα^−/−^ mice, the mice that received high-dose PFOA clustered separately from the other groups, indicating the marked effect of high-dose PFOA on hepatic gene expression.

Next, volcano plot analysis was performed to assess the magnitude of the effect of PFOA or GenX treatment on gene expression. In the wildtype mice, the largest effects were observed in the high-dose PFOA group (Figure 5B), with in total 788 genes significantly changed when applying a threshold of p ≤ 0.001 and fold-change >1.5 (489 up, 299 down; Figure 5D). Interestingly, while the overall effect of high-dose PFOA was substantially reduced in PPARα^−/−^ mice (Figure 5C), 294 genes were still significantly altered by high-dose PFOA in the absence of PPARα (207 up, 87 down; Figure 5E). Of the genes induced by high-dose PFOA in wildtype mice, 88% was dependent on PPARα. Compared to high-dose PFOA treatment, low-dose PFOA only induced limited changes in the hepatic transcriptome in both genotypes (Figure 5B–C). The overall effect of GenX on the hepatic transcriptome in the wildtype mice was intermediate between the high- and low-dose PFOA groups (79 up, 31 down; Figure 5D). Remarkably, no significant gene regulation by GenX was observed in livers of PPARα^−/−^ mice (1 up, 3 down; Figure 5E). Specifically, 99% of the genes induced by GenX in wildtype mice was regulated in a PPARα-dependent manner.

Next, we made a heatmap based on significantly regulated genes in the high-dose PFOA group compared to control-treated mice (Figure 5F). The figure illustrates that high-dose PFOA markedly impacted hepatic gene expression, which was attenuated—but still clearly visible—in the GenX and low-dose PFOA groups. Consistent with the other analyses, the effects of GenX on gene expression were completely abolished in the PPARα^−/−^ mice (Figure 5F), whereas the effects of low- and high-dose PFOA were strongly attenuated in the PPARα^−/−^ mice. Similar results were obtained when zooming in on the top 50 upregulated genes by high-dose PFOA (Figure 5G), many of which are well-established PPARα target genes [57]. Taken together, the gene expression data suggest that, (1) high-dose PFOA markedly influences hepatic gene expression, followed by GenX and low-dose PFOA, (2) the effects of PFOA on hepatic gene expression are predominantly mediated by PPARα, while the effects of GenX are entirely mediated by PPARα.

### 3.5 High-dose PFOA affects PXR and CAR signaling in the liver in the absence of PPARα

**Figure 6:**
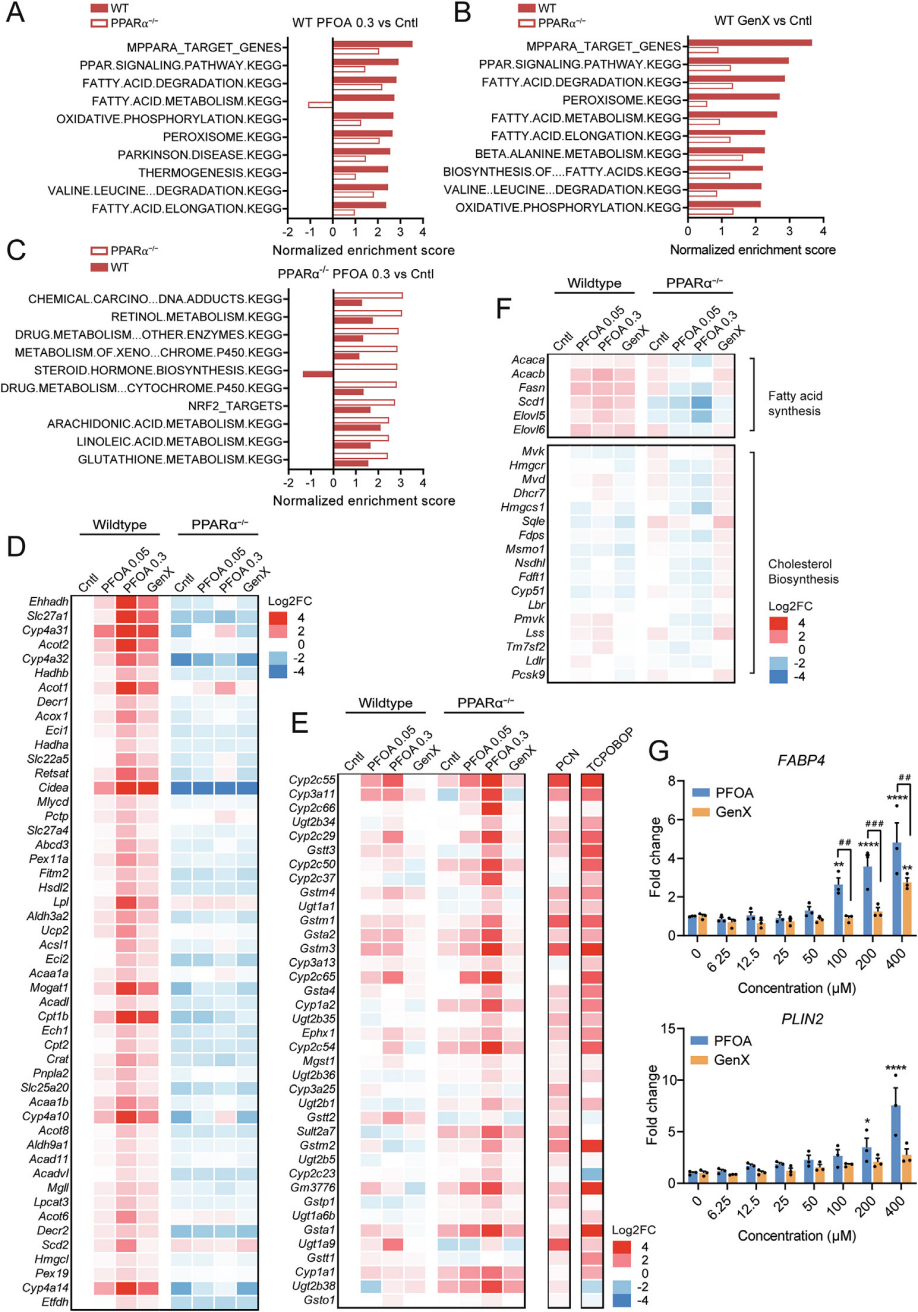
**Effects of PFOA in wildtype mice are mediated by PPAR**α. (A) Gene set enrichment analysis of the effect of 0.3 mg/kg bw/day PFOA vs control in livers of wildtype mice. The top 10 positively enriched gene sets in wildtype mice is shown, next to the normalized enrichment score for these gene sets in the PPARα^−/−^ mice. (B) Gene set enrichment analysis of the effect of GenX vs control in livers of wildtype mice. The top 10 positively enriched gene sets in wildtype mice are shown, next to the normalized enrichment score for these gene sets in the PPARα^−/−^ mice. (C) Gene set enrichment analysis of the effect of 0.3 mg/kg bw/day PFOA vs control in livers of PPARα^−/−^ mice. The top 10 positively enriched gene sets in PPARα^−/−^ mice are shown, next to the normalized enrichment score for these gene sets in the wildtype mice. (D) Heatmap of top 50 positively enriched genes belonging to the gene set mPPARα Target Genes. (E) Heatmap of positively enriched genes belonging to the gene set Chemical Carcinogenesis and DNA Adducts. Gene expression was compared to hepatic gene expression data of obese C57BL/6N mice treated with PCN, a selective murine PXR agonist (GSE136667), and C57BL/6N mice treated with the CAR-agonist TCPOBOP (GSE186654). (F) Heatmap of selected target genes of SREBP-1 and SREBP-2. (G) Gene expression profiles of *FABP4* and *PLIN2* in HepaRG cells treated with different concentrations of PFOA or GenX for 24 h. Data are expressed relative to control (DMSO 0.5%). Graphs are presented as mean ± SEM (n = 3 replicates). Asterisks indicate significant differences between treatment vs DMSO control (∗p < 0.05, ∗∗p < 0.01, ∗∗∗∗p < 0.0001). Hashtags indicate significant differences between PFOA vs GenX (^##^p < 0.01, ^###^p < 0.001).

Next, we aimed to get more insight into the functional impact of PFOA and GenX on biological pathways by performing gene set enrichment analysis (GSEA) (Figure 6A–C). As expected, pathways positively enriched by high-dose PFOA in wildtype mice were related to PPARα signaling, fatty acid metabolism, and oxidative phosphorylation (Figure 6A). Similar results were obtained for GenX (Figure 6B). Importantly, the enrichment scores for the PFOA-induced pathways were much lower in the PPARα^−/−^ mice, indicating strong PPARα dependency (Figure 6C). A heatmap with the top 50 positively enriched genes in the gene set mPPARα Target Genes is shown in Figure 6D, showing unequivocal PPARα-dependent gene regulation (total core enrichment of 117 out of 150 genes).

GSEA was also performed for high-dose PFOA in the PPARα^−/−^ mice. Significant positive enrichment was observed for pathways related to xenobiotic metabolism, steroid hormone biosynthesis, and omega-6 fatty acid metabolism (Figure 6C). Intriguingly, the enrichment scores for these pathways were much lower in the wildtype than PPARα^−/−^ mice, suggesting that the stimulation of xenobiotic metabolism and steroid synthesis by PFOA is attenuated by the presence of PPARα. A heatmap of the positively enriched genes in the gene set Chemical Carcinogenesis is shown in Figure 6E, showing stronger regulation by PFOA in the PPARα^−/−^ mice than in the wildtype mice. Many of these genes are significantly upregulated by the rodent-specific Pregnane X Receptor (PXR) agonist pregnenolone 16α-carbonitrile (PCN) (GSE136667) [58], as well as by the Constitutive Androstane Receptor (CAR) agonist TCPOBOP (GSE186654) [59], suggesting that they are PXR and CAR target genes.

Next to PPARα, Sterol Regulatory Element Binding Proteins have been linked to lipogenic (mainly SREBP-1) or cholesterogenic effects (mainly SREBP-2) of PFAS exposure [34,60,61]. Therefore, we assessed whether PFOA and GenX influenced SREBP-dependent gene regulation (Figure 6F). PFOA and GenX modestly induced the hepatic expression of SREBP-1 target genes involved in fatty acid synthesis, including *Acaca, Acacb, Fasn, Scd1, Elovl5* and *Elovl6* [62], whereas PFOA repressed the expression of these genes in PPARα^−/−^ mice. These data suggest PPARα-dependent upregulation and PPARα-independent downregulation of lipogenic genes by PFOA. Consistent with the PPARα specificity of GenX, no effects of GenX were observed in PPARα^−/−^ mice. PFOA slightly downregulated the expression of genes involved in cholesterol synthesis in wildtype and PPARα^−/−^ mice, suggesting PPARα-independent regulation [63].

Our qPCR and RNAseq data indicate that the effects of GenX on hepatic gene expression in mice are entirely mediated by PPARα. To examine if GenX also activates PPARα in human hepatocytes, we treated human HepaRG cells with GenX and studied the expression of the established PPARα target genes *FABP4* and *PLIN2*, which were selected based on their strong sensitivity to PPARα activation in HepaRG cells (Figure 6G) [64]. GenX dose-dependently stimulated the expression of *FABP4* and *PLIN2* in HepaRG cells yet was less potent than PFOA at the same concentration. These data suggest that GenX activates human PPARα, yet is a weaker agonist than PFOA.

## 4 Discussion

In this paper, we set out to study the impact of PFOA and GenX in a mouse model of diet-induced obesity, glucose intolerance, and NAFLD, and investigate the role of PPARα in mediating the metabolic effects of PFOA and GenX. Previous studies either examined the effect of PFOA in chow-fed mice or did not include PPARα^−/−^ mice. Our research reveals the major disruptive effects of PFOA and GenX on hepatic and systemic metabolism. Our main findings are: (1) high-dose PFOA improved glucose and insulin tolerance, which was independent of PPARα and likely related to reduced fat mass and body weight. (2) High-dose PFOA significantly reduced plasma triglycerides, cholesterol, NEFA, and glycerol, which except for triglycerides was independent of PPARα. (3) GenX and PFOA increased liver triglyceride levels in a PPARα-dependent manner. (4) The overall magnitude of transcriptome changes in the liver followed the order high-dose PFOA > GenX > low-dose PFOA. (5) 88% of the genes significantly induced by high-dose PFOA were regulated in a PPARα-dependent manner. For GenX, this was 99%, indicating that GenX is a more specific PPARα agonist than PFOA. (6) The PPARα independent effects of PFOA on hepatic gene expression are likely partially mediated by PXR and CAR.

Previous studies have shown that in vivo exposure to GenX leads to upregulation of PPARα target genes in rat and mouse livers, strongly suggesting that GenX is a PPARα agonist [36,37,65]. Importantly, we found that the effects of GenX on hepatic gene expression were completely abolished in PPARα^−/−^ mice, indicating that GenX acts exclusively via PPARα. Specifically, 99% of the upregulation of gene expression by GenX in mouse liver was dependent on PPARα. Equally high percentages of PPARα-dependent gene regulation were previously obtained for the highly specific PPARα agonists Wy-14,643 and fenofibrate [66,67]. This implies that the chemical contaminant GenX acts in the same way as certain hypolipidemic drugs used for lowering the risk of cardiovascular disease, although with a weaker agonistic effect. Importantly, GenX also upregulated the PPARα target genes *PLIN2* and *FABP4* in HepaRG cells, indicating that GenX is also an agonist of human PPARα.

Despite activating PPARα, GenX did not influence plasma glucose, cholesterol, triglycerides, glycerol, and NEFA. Previously, we and others observed a decrease in plasma triglycerides and NEFA and an increase in plasma cholesterol upon fenofibrate treatment [[68], [69], [70], [71], [72]]. The reason why these parameters were not changed upon GenX treatment is likely because of the relatively low dose used and because GenX is a comparatively weak PPARα agonist. Consistent with this notion, higher exposure levels of GenX were reported to lower plasma triglyceride levels in rat and mouse dams [65].

**Figure 7:**
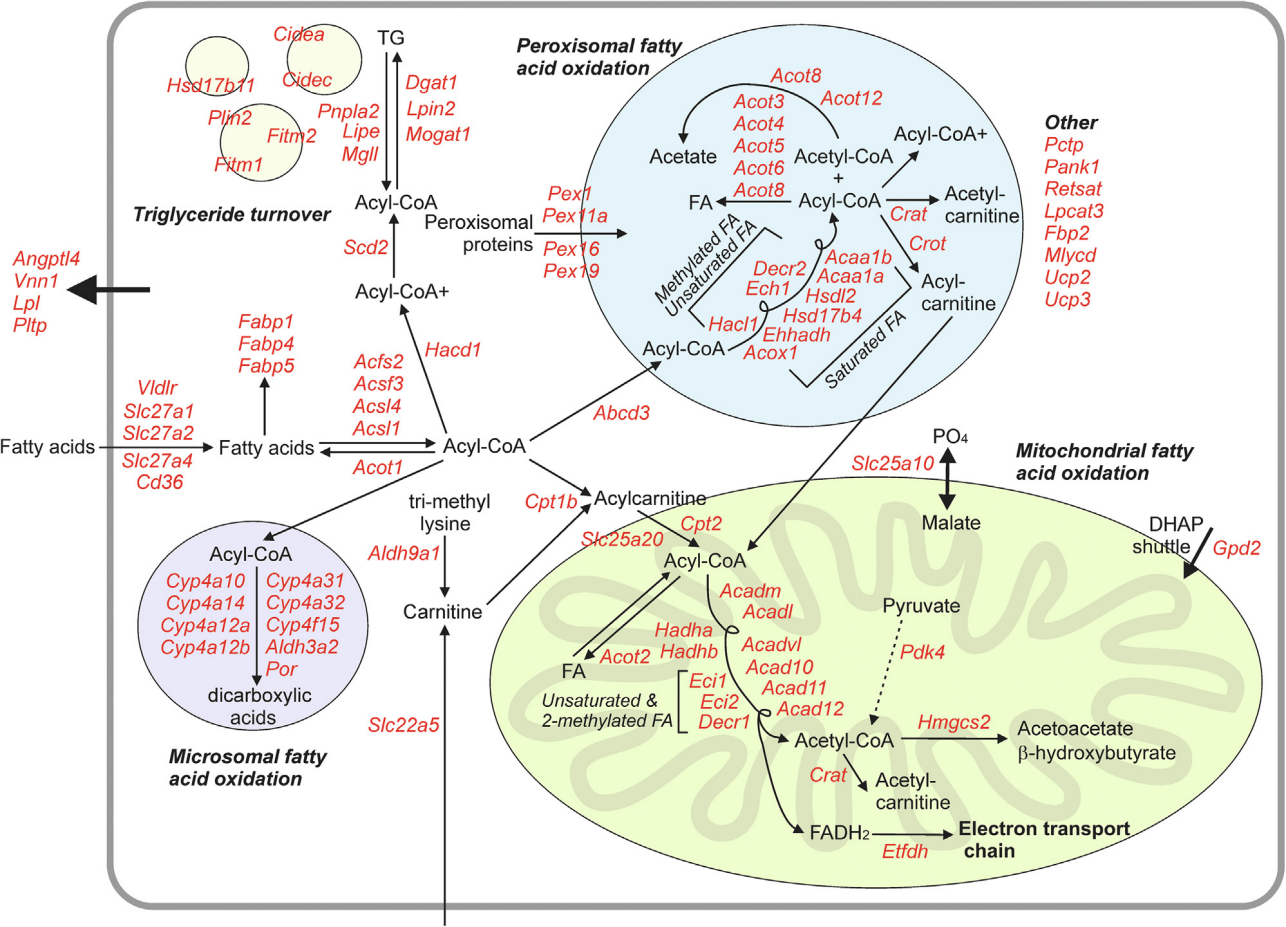
**Effect of PFOA on metabolic processes in mouse liver.** Manually constructed biochemical map of gene expression changes of PPARα-regulated genes (p < 0.001, fold change >1.5) after exposure to high dose PFOA for 20 weeks compared to control [57]. Genes in red indicate upregulation of expression.

Numerous studies have demonstrated that PFOA is a potent PPARα agonist in mice and humans [18,20,73]. In our study, most of the effects of high-dose PFOA on the liver transcriptome were abolished in PPARα^−/−^ mice. Specifically, 88% of the regulation of gene expression by high-dose PFOA in mouse liver was dependent on PPARα. This number is in line with data from Rosen et al., which showed ∼86% of PPARα-dependent gene regulation in 129S1/SvlmJ mice after 7 days of exposure to 3 mg/kg body weight/day of PFOA [67,74]. Interestingly, similar percentages of PPARα-dependent gene regulation in mouse liver were obtained for in vivo treatment with unsaturated fatty acids [66]. A transcriptome map was created of the PFOA-induced changes in expression of PPARα-regulated genes (Figure 7). The map illustrates the profound impact of PFOA on numerous PPARα-dependent pathways, including fatty acid uptake, binding, and activatio;, microsomal, peroxisomal, and mitochondrial fatty acid oxidation; ketogenesis, and triglyceride turnover.

Based on comparative analyses of hepatic gene regulation by the PXR and CAR agonists PCN and TCPOBOP, respectively, we deduced that the PPARα-independent gene regulation by PFOA is partially mediated by PXR and CAR. A role of PXR in mediating PPARα-independent gene regulation by PFOA has been previously recognized [21,23,67,75]. In addition, PFOA has been shown to activate CAR in different model systems [[21], [22], [23],^76^,^77^]. This is also evident in our model, in which the expression of the CAR target gene *Cyp2b10* was strongly upregulated by PFOA in both wildtype and PPARα^−/−^ mice. Since PXR and CAR are known to activate partly overlapping sets of genes, it is difficult to disentangle the separate roles of PXR and CAR in mediating the transcriptional effects of PFOA. Intriguingly, similar to observations by Rosen et al. [22], the induction of PXR/CAR targets by PFOA was more pronounced in PPARα^−/−^ mice than in wildtype mice. It can be speculated that this increased sensitivity to PXR/CAR activation in the PPARα^−/−^ mice might be due to a suppressive effect of PPARα on PXR and CAR, as crosstalk between nuclear receptors is known to exist [78]. However, so far there is no firm evidence supporting a suppressive effect of PPARα on PXR or CAR functioning. Alternatively, it is possible that in the absence of PPARα, more PFOA is available to bind and activate PXR/CAR.

In contrast to GenX, high-dose PFOA significantly decreased plasma triglycerides, cholesterol, and NEFA levels. Intriguingly, the suppressive effect of PFOA on plasma cholesterol was magnified in the PPARα^−/−^ mice. Taking into account the increased sensitivity to PXR activation in the PPARα^−/−^ mice and considering that PXR activation lowers plasma cholesterol, it can be speculated that PFOA reduces plasma cholesterol via PXR [79]. Concerning NEFA, plasma levels were decreased by high-dose PFOA in the wildtype and PPARα^−/−^ mice, suggesting that the effect is independent of PPARα. Since plasma NEFA levels are reportedly not affected by PXR activation [79], the reduction in plasma NEFA by PFOA is thus likely independent of PPARα and PXR. In contrast, the reduction in plasma triglyceride levels by high-dose PFOA was abolished in the PPARα^−/−^ mice, suggesting that this effect is dependent on PPARα. As indicated above, lowering of plasma triglycerides is a well-known therapeutic effect of synthetic PPARα agonists. Unlike PPARα activation, PXR activation does not seem to influence plasma triglycerides [79].

In contrast to rodent data, which generally show a reduction in plasma triglycerides and cholesterol upon PFAS treatment, epidemiological data mainly show positive associations between exposure to PFAS and total and LDL cholesterol and in some instances triglycerides [9,25,54]. A possible explanation for this apparent discrepancy is that PFAS may activate different receptors in mice and humans. Indeed, while PFOA potently activates both human and mouse PPARα, it only activates mouse PXR/CAR [20,21,34,73,76]. Accordingly, PXR/CAR are unlikely to be involved in mediating the biological effects of PFAS in humans. A conundrum remains, however, because rather than raising plasma triglycerides, PPARα activation in humans lowers plasma triglyceride levels. Accordingly, whether the association between PFAS exposure and serum lipid levels is causal remains unclear.

PPARα deficiency is known to be associated with elevated liver triglycerides [14,24,56,66,80,81], which we confirmed in our study. Intriguingly, liver triglycerides were also increased by PFOA or GenX treatment in a PPARα-dependent manner. The results are consistent with other studies showing that different PFAS increase hepatic triglyceride content in wildtype or humanized PPARα mice but not in PPARα^−/−^ mice [28,31,82]. It should be noted, though, that PFOA has also been reported to decrease hepatic triglyceride content in wildtype mice [32]. The latter study differed from ours in that PFOA was given to mice that had already received a high-fat diet for 16 weeks, suggesting that the effect of PFOA on hepatic triglyceride levels may depend on the nutritional/metabolic context. Since PPARα regulates hundreds of genes involved in hepatic lipid metabolism, including many genes involved in fatty acid oxidation, triglyceride storage, and lipolysis, it is difficult to pinpoint the exact mechanism underlying the effect of PFOA on hepatic triglycerides. Surprisingly, low-dose PFOA treatment significantly increased liver triglyceride content but not liver weight or plasma parameters. Although the changes in hepatic gene expression triggered by low-dose PFOA were modest, heatmaps revealed similar trends in expression patterns between low- and high-dose PFOA treatment. This suggests that low-dose PFOA induced relatively subtle expression changes, which upon chronic exposure could lead to more drastic effects, such as increased liver triglycerides. Such a scenario is not unrealistic, as PFAS are highly persistent and bio-accumulate in the body, leading to chronic exposure.

SREBPs have been implicated in the effects of PFAS [21,60,61]. In the current study, genes involved in de novo lipogenesis, which are under transcriptional control of SREBP-1, were induced in response to PFOA and GenX. Hence, the triglyceride accumulation in livers of PFOA and GenX-treated mice may be partly explained by increased lipogenesis. Remarkably, we found a downregulation of lipogenic genes by PFOA in the PPARα^−/−^ mice, suggesting crosstalk between SREBP-1 and PPARα, likely involving an upregulation of SREBP-1 by PPARα [62,83]. Accordingly, the stimulation of lipogenic genes by PFOA and GenX might be due to an indirect activation of SREBP-1 via PPARα. Next to SREBP-1, studies also reported the effects of PFOA on SREBP-2 signaling and cholesterol biosynthesis [24,34]. In our study, we only observed a marginal downregulation of SREBP-2 target genes in response to PFOA.

An interesting and seemingly paradoxical observation is that both PPARα activation by PFOA and GenX as well as PPARα deficiency led to higher hepatic triglyceride levels. It should be noted, though, that the effects of PPARα activation and deficiency on liver phenotype do not necessarily have to be the opposite. Whereas some genes are strongly induced by PPARα activation but minimally affected by PPARα deficiency, other genes are minimally induced by PPARα activation but strongly suppressed by PPARα deficiency [13]. Depending on the specific role of the altered genes in fatty acid catabolism and triglyceride synthesis/storage, PPARα activation and deficiency might both increase hepatic triglyceride levels, albeit via different mechanisms.

Limited research has been conducted on the effect of PFAS on triglyceride levels in the human liver. Recently, Sen et al. found a positive association between PFAS concentrations in serum and NALFD-associated lipid changes in the livers of humans [84]. In human HepaRG cells, high concentrations of PFOA, PFOS, and PFNA increased triglyceride content [34]. By contrast, in primary human hepatocytes, PFOA and PFOS did not significantly alter triglyceride content [85]. Further study on the impact of PFAS on triglyceride content in the human liver is warranted, for example, in hepatocyte humanized mice [86,87].

One of the strengths of the current study is that we exposed mice to relatively low doses of PFOA and GenX. Specifically, the exposure levels of the high- and low-dose PFOA treatment were 10- and 60-fold lower, respectively, than used in most studies. Nevertheless, they still exceed expected human exposure levels based on current intake via food, drinking water, or other sources [88]. For instance, the maximum upper boundary of intake for the group with the highest risk of PFAS exposure—based on the sum of the most common types of PFAS (PFOA, PFOS, PFNA, PFHxS)—is currently estimated at 96 ng/kg body weight per week [7]. The lowest PFOA concentration applied in the current study would lead to an exposure of 350 μg/kg body weight/week and is thus several orders of magnitude higher. Nevertheless, high variability in PFAS levels in foods exists, which creates a large uncertainty in determining true exposure levels. Indeed, people living in high-risk areas are likely exposed to much higher concentrations of PFAS, with concentrations in drinking water reaching up to 1475 ng/L for PFOA alone [8,89,90]. Moreover, humans are usually exposed to a mixture of different types of PFAS, which could result in higher total PFAS levels.

Our study also has limitations. First, we found a significant decrease in food intake after treatment with PFOA and GenX, which might impact certain metabolic parameters. The reduced food intake is in line with findings from other PPARα agonists, such as oleoylethanolamide, GW-7647, and fenofibrate [[51], [52], [53]]. It should be noted, though, that except for the high-dose PFOA group, the reduction in food intake was not accompanied by a decrease in body weight. Second, we found a significant improvement in glucose and insulin tolerance in wildtype and PPARα^−/−^ mice exposed to high-dose PFOA. However, it is conceivable that the improved glucose homeostasis is a mere reflection of the lower fat mass and thereby body weights in these mice rather than a direct effect of PFOA on glucose homeostasis. Third, we used mice that were deficient in PPARα in all tissues rather than just in the hepatocytes. As PPARα is well expressed in the heart, muscle, intestine, and brown adipose tissue [91,92], it is possible that part of the effects of PFOA and GenX may be conveyed by PPARα in extra-hepatic tissues.

In conclusion, our results show dose-dependent disturbances in hepatic lipid metabolism by PFOA in mice. At a relatively low dose, PFOA increased hepatic triglyceride levels. The metabolic and transcriptomic effects of PFOA were mainly mediated by PPARα, although the involvement of PXR and CAR was also evident. Compared to PFOA, GenX was found to be a less potent but more specific PPARα activator that also raised hepatic triglyceride levels. Our data thus justify the concern about the disruptive effect of PFAS on hepatic and systemic metabolism and stress the need for regulation of these chemicals.

## Author contributions

B.A. and S.K. conceived and planned the research and experiments. B.A. carried out the mouse study and performed the experiments. B.A. and S.K. analyzed the data. G.H. performed the RNA sequencing analysis. D.R. and A.J. performed the HepaRG experiments. E.M.v.S contributed to the interpretation of the results. B.A. performed the statistical analyses. B.A. and S.K. wrote the manuscript. All authors provided critical feedback and helped to shape the research, analysis, and manuscript.

## Data Availability

Data will be made available on request.
